# Towards comprehensive characterization of Cs-137 Seeds using PRESAGE® dosimetry with optical tomography

**DOI:** 10.1088/1742-6596/444/1/012100

**Published:** 2013

**Authors:** J Adamson, Y Yang, L Rankine, J Newton, J Adamovics, O Craciunescu, M Oldham

**Affiliations:** 1 Dept. of Radiation Oncology, Duke University Medical Center Box 3295, Durham NC 27710; 2 Dept. of Chemistry, Rider University, Lawrenceville, NJ 08648

## Abstract

We describe a method to directly measure the radial dose and anisotropy functions of brachytherapy sources using polyurethane based dosimeters read out with optical CT. We measured the radial dose and anisotropy functions for a Cs-137 source using a PRESAGE® dosimeter (9.5cm diameter, 9.2cm height) with a 0.35cm channel drilled for source placement. The dosimeter was immersed in water and irradiated to 5.3Gy at 1cm. Pre- and post-irradiation optical CT scans were acquired with the Duke Large field of view Optical CT Scanner (DLOS) and dose was reconstructed with 0.5mm isotropic voxel size. The measured radial dose factor matched the published fit to within 3% for radii between 0.5–3.0cm, and the anisotropy function matched to within 4% except for *θ* near 0° and 180° and radii >3cm. Further improvements in measurement accuracy may be achieved by optimizing dose, using the high dynamic range scanning capability of DLOS, and irradiating multiple dosimeters. Initial simulations indicate an 8 fold increase in dose is possible while still allowing sufficient light transmission during optical CT. A more comprehensive measurement may be achieved by increasing dosimeter size and flipping the source orientation between irradiations.

## 1. Introduction

Accurate and comprehensive measurement of dose distributions about brachytherapy sources remains a challenging yet relevant problem. Varying dose rates and complex distributions about these sources require a measurement system capable of high spatial accuracy and resolution, and large dynamic range [1]. Due to these challenges, most published characterizations of brachytherapy sources rely heavily on Monte Carlo simulations with experimental verification at select points [2]. 3D dosimetry has potential to provide a more comprehensive verification. A number of studies have investigated gel dosimetry for brachytherapy [3, 4]; sensitivity of the polymerization process to oxygen, energy dependence (for low energy sources), and diffusion of monomers across steep dose gradients close to the source are challenges to achieving accurate results [1]. Another 3D dosimeter, PRESAGE® (Heuris Inc, NJ) [3, 5], is a polyurethane based dosimeter that is solid and transparent. Some of its advantages include being machinable, moldable, insensitive to oxygen exposure, and having minimal signal diffusion, which are important for brachytherapy. PRESAGE® has been shown to be tissue equivalent for Ir-192 and Cs-137. These dosimeters can be analyzed using optical CT, one system of which is the Duke Large field of view Optical CT-Scanner (DLOS) [6]. This scanner has minimal scatter and is capable of reconstructing PRESAGE® distributions with sub-millimeter voxel size.

This study we investigate the feasibility of using PRESAGE®/DLOS to investigate dose distributions about a Cesium-137 source [7]. Specifically we describe initial attempts to directly calculate and verify Monte Carlo based parameters from the formalism developed by the American Association of Physicists in Medicine (AAPM) Task Group 43 (TG-43) [2].

## 2. Materials & Methods

### 2.1. Measurement

A cylindrical PRESAGE® dosimeter [8] was obtained (9.5cm diameter) with a 0.35cm diameter channel drilled in the center to midline. An optical CT pre-scan was acquired using DLOS [6] in which 720 projection images were acquired at 0.5° increments in ~15 minutes. The Cs-137 source [7] was inserted into the channel for irradiation and 5.3Gy was delivered at 1cm with the dosimeter placed in water to provide backscatter (40 cm)^3^. After irradiation a post optical CT was acquired and the dose distribution reconstructed with (0.5 mm)^3^ isotropic voxel size and no median filtering.

### 2.2. TG-43 Parameters

In TG-43, the dose rate constant, Λ, radial dose function, *g_L_(r)*, and anisotropy function, *F(r, θ)*, are parameters that are specific to each source model. We extracted the radial dose and anisotropy functions from the measured 3D dose distribution. Given the source location and orientation, the radius *r* and angle *θ* can be calculated for each voxel of the measured distribution.

We calculate a fit, *ĝ_L_*(*r*), of the radial dose function from the measured distribution which includes all voxels within ±0.1° or ±0.5mm of the transverse plane. A 5-degree polynomial fit is used to model *g*_L_(*r*), where x-values in the fit are the radius *r* per voxel, and y-values are:

(1) $$y_{i}=\frac{I(r_{i},\theta_{0})}{\bar{I}(r_{0},\theta_{0})}\frac{G_{L}(r_{0},\theta_{0})}{G_{L}(r_{i},\theta_{0})}$$

where *G_L_(r, θ)* is the geometry function, *I*(*r_i_*,*θ_0_*) is the intensity accumulated in voxel *i* of the 3D distribution and *Ī*(*r_0_*,*θ_0_*) is the mean intensity for all voxels within ±0.1° and ±0.5mm of *θ_0_* and *r_0_*.

We calculated the anisotropy function out to the max distance measurable for our dosimeter. At a given *r_j_* and *θ_k_*, we calculate the anisotropy function using voxels in the dosimeter within ±0.5° and ±1.0mm with the equation:

(2) $$\hat{F}(r,\theta )=\sum_{i=1}^{n}\frac{I(r_{i},\theta_{i})}{G_{L}(r_{i},\theta_{i})}\frac{G_{L}(r_{0},\theta_{0})}{\bar{I}(r_{0},\theta_{0})\cdot {\hat{g}}_{L}(r_{i})}$$

where *ĝ_L_*(*r_i_*) is calculated using the polynomial fit described above.

### 2.3. Data Preparation

Preparatory steps prior to calculating the radial dose and anisotropy functions included defining the region of interest (ROI) within the dosimeter and registering the source location and orientation. The ROI excluded the channel and voxels with zero or negative signal. Finally a morphological erosion algorithm [9] excluded voxels near imperfections and the dosimeter surface. The source location and orientation was determined by iteratively calculating *ĝ_L_*(*r*) and *F̂_L_*(*r*, *θ*) for different source locations and orientations and minimizing a score function that was the product of their residual errors.

## 3. Results

**Figure 1:**
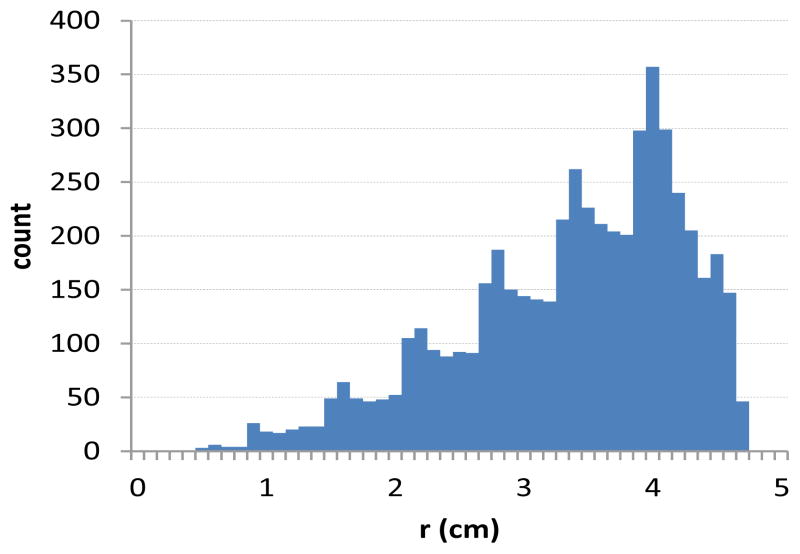
Number of measurement points along the transverse plane with 1mm binning.

**Figure 2:**
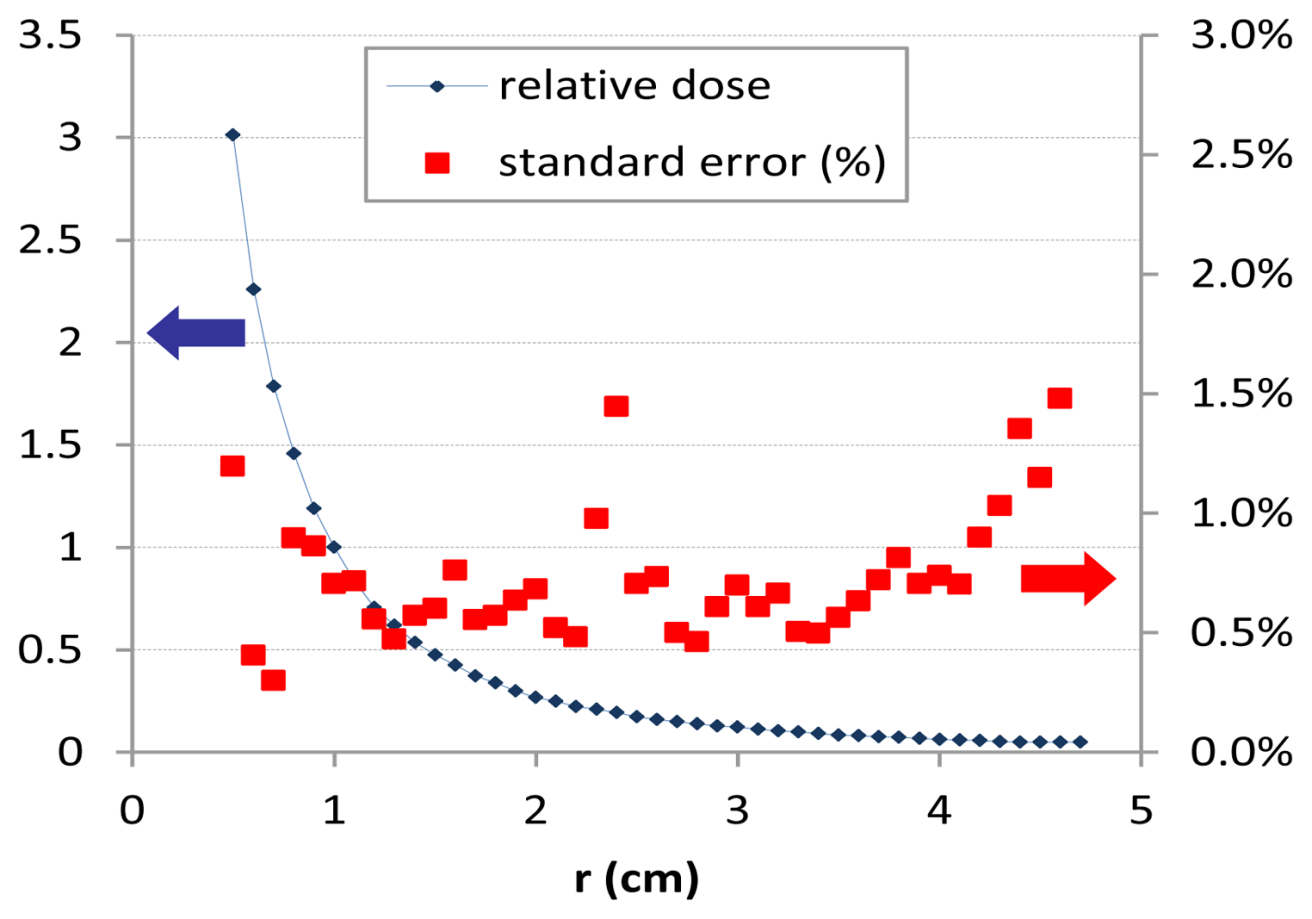
Relative dose & standard error along transverse plane. Diminishing dose is offset by increasing sample size (Fig. 1) resulting in nearly constant standard error.

**Figure 3:**
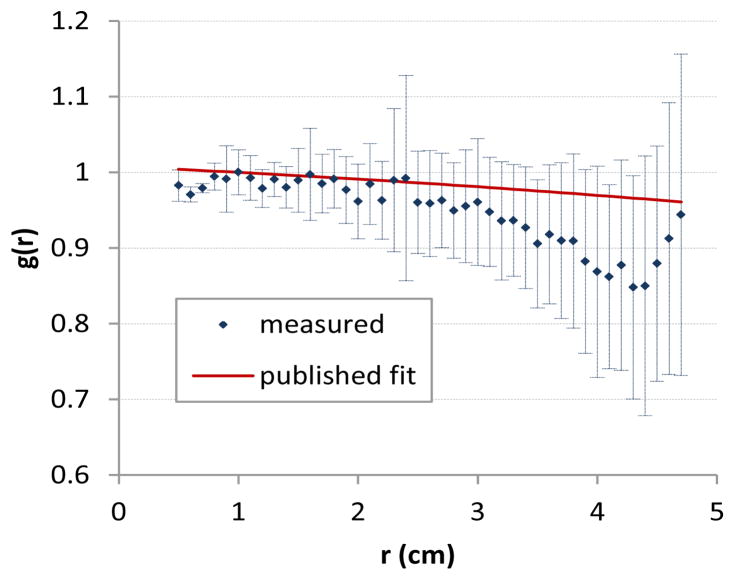
Radial dose function in 1 mm increments measured using PRESAGE® / optical CT and the published fit from Monte Carlo simulations [7].

Figures 1 and 2 describe the data along the transverse plane defining the radial dose function, and illustrate an advantage of 3D dosimetry. As *r* increases, so does the sample size of voxels, resulting in a nearly constant standard error. The radial dose function is shown in Figure 3 where data was binned in 1mm increments and error bars represent one standard deviation. The factor matched the published fit to within 3% for radii of 0.5–3.0cm. Beyond 3cm the radial dose function was underestimated by as much as 10%. This underestimation is a topic of current investigation and may be attributed to differences in density or the edge of the dosimeter.

Table 1 shows the difference from the published anisotropy function. The function matched to within 4% except when *θ* approached 0° and 180° and radii > 3cm.

## 4. Discussion

We have investigated feasibility of measuring the radial dose and 2D anisotropy functions for a Cs-137 source using PRESAGE®/Optical CT. One advantage shown here for 3D dosimetry is that low signal at distance is offset by increased number of voxels. Measurement of TG-43 parameters using 3D dosimetry will allow for direct comparison to the factors calculated by Monte Carlo, has potential to allow for investigation of the uncertainty in the various factors. TG-43 states: “sufficient data are not available…to provide a comprehensive uncertainty analysis for anisotropy functions. More research is needed… identifying geometric parameters to which (the anisotropy function) is sensitive” [2]. This is an evident area where source characterization using 3D dosimetry will be of use.

The following options remain to be investigated to optimize source characterization using PRESAGE®/Optical CT:

- Optimizing delivered dose: A tradeoff must be made between achieving acceptable signal to noise ratio distal from the source and achieving sufficient light transmission through the dosimeter during optical CT acquisition. ScanSim is a tool for simulating optical CT imaging [10] which we intend to use to determine the maximum dose that still achieves sufficient light transmission during optical CT with DLOS. Initial estimates indicate an optimal attenuation coefficient of 0.95cm^−1^ at 1cm, corresponding to a dose of ~8Gy at 1cm for a high sensitivity dosimeter.
- High Dynamic Range Scanning: DLOS can be operated in a “dual gain” mode where two acquisitions are performed with varied parameters [11]. In this mode a second scan is performed where the light source intensity is increased, which increases sensitivity at high optical densities.
- Increasing dosimeter size: Larger dosimeters have been used in conjunction with DLOS and can be implemented in future work.
- Using multiple irradiations: Systematic errors can be minimized using irradiation of multiple dosimeters. Also the channel in the dosimeter center results in missing data for the anisotropy function which can be overcome by using multiple irradiations with reversed source orientation.
- Determining dose rate constant: This is the only source model specific parameter that was not measured. Each dosimeter is accompanied by cuvettes used to quantify dosimeter sensitivity and in theory could be used to measure the dose rate constant.

## Figures and Tables

**Table 1:** Measured 2D anisotropy function for a Cs-137 source [7]. Shown is the percent difference from published fit ± standard error (# measurement voxels).

theta (°)	r (cm)					
	0.75	1	1.5	2	3	4
10	-	-	-	-	4.7±4.3% (16)	-
20	-	-	-	−6.6±2.9% (24)	−7.4±1.9% (45)	-
30	-	-	−0.3±2.0% (15)	−3.8±1.8% (58)	0.9±1.3% (86)	-
40	-	-	−1.6±1.2% (21)	−2.5±1.0% (62)	−3.1±0.9% (107)	−0.2±0.8% (266)
50	-	−0.7±1.6% (22)	−0.7±0.7% (29)	−0.3±0.7% (72)	−1.4±0.6% (138)	−4.0±0.7% (312)
60	-	0.7±1.1% (18)	−0.5±0.6% (46)	−1.5±0.5% (89)	−1.7±0.6% (149)	−2.0±0.6% (351)
70	-	1.3±0.8% (21)	−1.1±0.5% (48)	−1.8±0.5% (84)	−1.7±0.5% (179)	−3.5±0.6% (354)
80	1.0±0.8% (12)	0.9±0.6% (30)	−1.3±0.5% (28)	−0.5±0.5% (95)	−0.1±0.4% (142)	0.5±0.6% (382)
90	-	1.8±0.3% (13)	0.2±0.3% (66)	−0.8±0.4% (128)	0.4±0.4% (141)	1.1±0.6% (365)
100	−0.1±0.4% (11)	1.5±0.3% (33)	0.6±0.4% (29)	−0.6±0.4% (87)	−0.4±0.4% (145)	−1.1±0.5% (395)
110	-	2.4±0.4% (24)	1.0±0.5% (47)	−0.4±0.4% (80)	0.0±0.3% (173)	−2.0±0.5% (361)
120	-	1.2±0.7% (17)	0.5±0.4% (39)	−0.6±0.3% (99)	−0.8±0.2% (140)	−2.9±0.4% (351)
130	-	4.1±1.0% (18)	0.7±0.6% (28)	−0.8±0.2% (70)	−1.0±0.2% (155)	−4.1±0.3% (304)
140	-	-	1.8±0.3% (24)	−0.2±0.2% (54)	−4.0±0.3% (113)	−4.9±0.3% (251)
150	-	-	2.5±0.3% (17)	−0.1±0.2% (57)	−3.2±0.6% (84)	−4.7±0.3% (191)
160	-	-	2.6±0.2% (11)	−0.2±0.2% (34)	−2.2±0.2% (55)	−4.9±0.4% (132)
170	-	-	-	−1.6±0.3% (8)	−3.6±0.4% (31)	−4.6±0.6% (72)
172	-	-	-	−1.5±0.3% (12)	−3.9±0.4% (30)	−9.4±0.7% (54)
174	-	-	-	-	−1.8±0.9% (17)	−6.8±1.4% (40)
175	-	-	-	-	−1.7±0.5% (17)	−4.6±1.5% (32)
176	-	-	-	-	−2.6±0.4% (16)	−3.2±1.6% (27)
